# Carbon assimilation through a vertical light gradient in the canopy of invasive herbs grown under different temperature regimes is determined by leaf and whole-plant architecture

**DOI:** 10.1093/aobpla/plaa031

**Published:** 2020-06-28

**Authors:** Andreas Jorgensen, Brian K Sorrell, Franziska Eller

**Affiliations:** Department of Bioscience, Aarhus University, Aarhus C, Denmark

**Keywords:** Horizontal leaves, *Iris pseudacorus*, lanceolate leaves, light response curves, *Lythrum salicaria*, maximum light-saturated photosynthesis rate, N allocation, photosynthetic pigments

## Abstract

This study examined the acclimation to temperature of two globally invasive species *Iris pseudacorus* and *Lythrum salicaria*, which share the same habitat type but differ in morphology. *Iris pseudacorus* has long vertical leaves, allowing light penetration through the canopy, while *L. salicaria* has stems with small horizontal leaves, creating significant self-shading. We aimed to build a physiological understanding of how these two species respond to different growth temperatures with regard to growth and gas exchange-related traits over the canopy. Growth and gas exchange-related traits in response to low (15 °C) and high (25 °C) growth temperature regimes were compared. Plants were grown in growth chambers, and light response curves were measured with infrared gas analysers after 23–33 days at three leaf positions on each plant, following the vertical light gradient through the canopy. After 37 days of growth, above-ground biomass, photosynthetic pigments and leaf N concentration were determined. The maximum photosynthesis rate was lower in lower leaf positions but did not differ significantly between temperatures. *Iris pseudacorus* photosynthesis decreased with decreasing leaf position, more so than *L. salicaria*. This was explained by decreasing N and chlorophyll concentrations towards the leaf base in *I. pseudacorus*, while pigment concentrations increased towards the lower canopy in *L. salicaria.* Biomass, shoot height and specific leaf area increased with temperature, more so in *I. pseudacorus* than in *L. salicaria.* Light response curves revealed that *L. salicaria* had a higher degree of shade acclimation than *I. pseudacorus*, probably due to self-shading in *L. salicaria*. High temperature decreased C assimilation at the bottom of the canopy in *L. salicaria*, while C assimilation in *I. pseudacorus* was less affected by temperature. As vegetative growth and flowering was stimulated by temperature, the invasive potential of these species is predicted to increase under global warming.

## Teaser

Photosynthetic carbon gain throughout the canopy was affected differently by temperature in the invasive yellow flag iris (*Iris pseudacorus*) and purple loosestrife (*Lyhtum salicaria*). While unaffected in *I. pseudacorus*, lower canopy photosynthesis decreased with high temperature in *L. salicaria*. *Lythrum salicaria* showed typical shade-acclimated responses in leaf pigment content and light response curves which were attributed to adaptations to self-shading. *Iris pseudacorus* did not show any such adaptations, revealing different invasive photosynthetic strategies in the two species. Overall, both species benefited from elevated temperatures with increased biomass and shoot height as well as flowering rate in *L. salicaria*.

## Introduction

Invasive plant species negatively affect native species diversity and ecosystem functioning (Mack *et al.* 2000; Gritti *et al.* 2006). After novel species are introduced to new habitats by human activity, natural species distribution patterns can be altered, causing potentially severe changes to ecosystem dynamics. As climatic conditions are a major constraint on the distribution of plant species, climate change can also be a powerful driver of both native and invasive species distributions (Walther *et al.* 2009; Moran and Alexander 2014; Fernández de Castro *et al.* 2018). Climate warming is likely to shift habitats to higher latitudes and elevations (IPCC 2014), which could promote invasions by species already adapted to higher temperatures, ultimately shifting species distributions globally.

Invasive plants are often characterized by high productivity and effective dispersal, enabling them to out-compete less productive native species and dominate ecosystems where they are introduced. Invasive strategies for maintaining rapid dispersal include, but are not limited to, large numbers of seeds produced, high seed viability, dispersal by fragmentation and spread by rhizomes or, as is often the case, combinations of these (Thompson *et al.* 1987; Gaskin *et al.* 2016). Furthermore, productivity is generally higher at high temperatures since metabolic activity increases with temperature (Berry and Bjorkman 1980). The positive relationship between temperature and productivity favours already highly productive species (Daehler 2003), enhancing their capacity for rapid growth and resource competition. Morphology can also be important in determining the invasive success of a species, since morphological traits such as leaf or root system architecture greatly affect the ability to utilize resources such as nutrients and light energy (Wells *et al.* 1986; Comas *et al.* 2013).

With temperatures rising globally and invasions likely becoming more frequent, it is increasingly important to understand physiological responses of invasive plants to warmer temperatures. Our study focuses on purple loosestrife (*Lythrum salicaira*) and yellow flag iris (*Iris pseudacorus*), two herbaceous wetland species native to Europe that are highly invasive on other continents. *Lythrum salicaria* behaves invasively in USA and Canada (Blossey *et al.* 2001; Hovick *et al.* 2011), while *I. pseudacorus* has been formally classified as a weed in the USA (USDA 2013), Canada (Forest Operations, Lands & Natural Resource 2019), Japan (Ministry of the Environment 2005), New Zealand (Ministry for Primary Industries 2017) and South Africa (Department of Environmental Affairs 2016). Both species are common in water-saturated soils in wetlands and adjacent to lakes, ponds, rivers or streams in temperate and subtropical regions (Mal *et al.* 1992; Jacobs *et al.* 2011). In Europe, both species have natural ranges from Norway to southern Spain (Barkley *et al.* 1980; Thompson *et al.* 1987). This large latitudinal gradient means that both species can persist across a relatively wide thermal range, which is indicative of some degree of thermal plasticity in both species. These two species provide an interesting subject for invasion biology, because they grow under similar environmental conditions, yet they have distinctly different morphologies, providing two seemingly successful adaptive strategies for colonizing freshwater wetland habitats.

The aim of this study was therefore to understand how the two globally invasive species *I. pseudacorus* and *L. salicaria* respond to different growth temperatures in a comparative analysis of growth and gas exchange-related traits, as well as pigment, nitrogen and carbon content. *Lythrum salicaria*, a perennial eudicot, has multiple woody stems with numerous side branches. Both stems and side branches have large numbers of relatively small horizontal leaves (Mullin 1998). This growth form creates a significant amount of self-shading, especially in the lower parts of the canopy. Self-shading does not merely attenuate the incoming light quantity; it also results in light scattering or diffuse light. Diffuse light can result in lowered photosynthesis rates even at similar total irradiance (Brodersen *et al.* 2008; Urban *et al.* 2014; Earles *et al.* 2017). The shading effects may therefore be amplified by diffuse lighting, although the response seems to be species-specific and driven by leaf biochemical traits (Berry and Goldsmith 2019). Although assimilation rates are expressed on an area basis, total leaf area does not necessarily equal plant growth, as C partitioning between organs can differ and lead to significant changes in plant growth (Weraduwage *et al.* 2015). While a large leaf area index (LAI) allows the plant to harvest large amounts of light energy per unit ground area, respiration may increase in certain plant species due to the larger amount of shaded leaf area, lowering net C assimilation (Bunce 1989). As respiration generally increases faster with temperature than photosynthesis (Berry and Bjorkman 1980), we hypothesized that *L. salicaria* is better adapted to cooler growth temperatures where C loss due to respiration is limited. Furthermore, since *L. salicaria* is a C3 plant, both photosynthesis and photorespiration are expected to increase with temperature, which means that even though the photosynthesis rate (*A*) is likely to increase with temperature, a smaller increase in C assimilation is expected due to photorespiratory C loss. These conditions favour photosynthesis at low temperatures.

*Iris pseudacorus*, a perennial monocot, has long vertical leaves (up to 1 m) produced directly from the base of the plant. This leaf architecture minimizes self-shading and thereby reduces daytime respiration from shaded leaves (Jespersen *et al.* 2017). A high maximum light-saturated photosynthesis rate (*A*_max_) is expected throughout the plant since most of the leaf area is exposed to high light intensities. However, prolonged exposure to very high light intensities can cause photoinhibition, primarily because of damage to photosystem II (PSII), which decreases photosynthesis rate (Powles 1984; Aro *et al.* 1993). Photoinhibition is often more severe at low temperatures (Long and Humphries 1994; Takahashi and Murata 2008) because of a slower repair of PSII due to lower activity of repair enzymes at low temperatures (Takahashi and Murata 2008). We therefore hypothesize that *I. pseudacorus* is best adapted to warm growing temperatures where photoinhibition is limited.

With light intensity decreasing through the canopy, a decreasing *A*_max_ from the top to the bottom of the plant is expected for both species. As light attenuation through the canopy is likely to be significantly greater in *L. salicaria* than in *I. pseudacorus* due to greater self-shading, a greater decrease in assimilation rates is expected in *L. salicaria.*

## Materials and Methods

### Plant material and experimental set-up

Seeds of *L. salicaria* and *I. pseudacorus* were germinated in a greenhouse in early August 2018. *Lythrum salicaria* seeds were commercial seeds from Denmark. *Iris pseudacorus* seeds were taken from a Danish population which had been grown in a greenhouse for at least 2 years (originally collected in the wild). On 27 August seedlings were brought to the growth facilities at Aarhus University. Each seedling was placed in a 1.5-L pot and peat soil was used as the growth substrate. Eight replicates of each species were placed on the floor in 4.9 m^2^ growth cambers (Karl Weis, Berlin, Germany) at 15 °C (low-temperature treatment) and 25 °C (high-temperature treatment) during the light period. The temperature regimes were chosen to differ greatly to cause detectable acclimation responses, but also to lie within the natural range of the species’ experienced growth temperatures. Seedlings from each species were of similar height at the beginning of the experiment. Mean height of *I. pseudacorus* seedlings was 57.2 ± 5.3 cm while *L. salicaria* seedlings measured 50.3 ± 17.4 cm.

The growth chamber settings were as follows: light intensity at 1 m above the floor was 400 μmol m^−2^ s^−1^ PAR (photosynthetic active radiation) using Phillips GP-E 600-W 400-V light sources. Relative humidity was ~75 %. The day-night cycle was 16 h of light and 8 h of darkness and night temperatures were 2 °C lower than the day temperatures. Twice weekly each plant was given 100 mL of a nutrient solution consisting of 500 mg L^−1^ ‘Pioner NPK Makro 19-2-15 + Mg (Green)’ and 0.05 mL L^−1^ ‘Pioner Mikro Plus with Iron’ (Horticoop Scandinavia). Nutrient concentrations are listed in Table 1. The pH of the nutrient solution was adjusted with sulfuric acid to ~6.5 in order to maximize nutrient uptake by the plants. The plants were watered approximately daily as needed. Twice weekly the plants were rotated within the chamber in order to avoid effects of environmental gradients inside the chamber. The species were not mixed but grouped separately within the chamber to avoid shading effects, especially in the *I. pseudoacorus* canopy. The plants were grown in the chambers for 37 days until they were harvested on 2 October.

**Table 1.** Nutrient concentrations used during the experiment. *Iris pseudacorus* and *Lythrum salicaria* grown at 15 °C and 25 °C received 100 mL of the solution twice per week.

Macronutrient	Concentration (mg L^−1^)	Micronutrient	Concentration (%)
NO_3_-N	59.5	B	0.32
NH_4_-N	37.0	Cu	0.13
Total-N	96.5	Fe	1.62
P	11.5	Mn	0.63
K	77.0	Mo	0.06
Mg	15.0	Zn	0.32
S	19.5	–	–

### Growth parameters

For each plant, shoot height and above-ground biomass were determined on the final day of the treatment. For *I. pseudacorus*, height was measured from the soil surface to the tip of the longest leaf. For *L. salicaria*, height was measured from the soil surface to highest point of the plant, either a leaf or inflorescence. The plants were then harvested and the above-ground biomass determined after drying in an oven at 80 °C for 48 h, followed by drying at 60 °C for 72 h. [see Supporting Information—File S1]

### Photosynthetic parameters

Three leaf positions on each plant were marked. On *L. salicaria*, leaf positions represented distinct leaves positioned along the vertical light gradient through the canopy. Leaves were clustered partly within each other in *I. pseudacorus*; hence, leaf positions were chosen along the same, outer leaf to obtain measurements from the entire vertical light gradient. The leaf positions were denoted as upper (top of plant), middle (middle of plant) and lower (bottom of plant) position.

Photosynthetic light response curves were obtained after 23–33 days of temperature treatment. *Lythrum salicaria* was measured between 18 and 21 September and *I. pseudacorus* was measured between 24 and 28 September. Measurements were made between 0800 and 1800 h. Light response curves were obtained using two LI-6800 portable photosynthesis systems with 6800-02 LED light sources (Li-COR Biosciences, Lincoln, NE, USA). Of these, one was used in the low-temperature chamber and the other in the high-temperature chamber. Pilot measurements under similar environmental conditions showed that measuring differences between the two machines was negligible. A built-in automatic program for light response curves was used with a minimum waiting time of 90 s and a maximum waiting time of 180 s between each measurement. Before the curve was started, gas exchange rates were allowed to stabilize at the highest light intensity. The photosynthesis rate was then measured sequentially at 2000, 1500, 1000, 500, 250, 120, 60, 30, 15 and 0 μmol m^−2^ s^−1^ PAR. Relative air humidity was set to 55 % and air flow was set to 500 μmol s^−1^. The leaf chamber CO_2_ concentration was set at 400 μmol mol^−1^ with carbon dioxide provided from an external CO_2_ cartridge. The leaf temperature was set to 16 °C in the low-temperature treatment and 26 °C in the high-temperature treatment. The set leaf temperatures were chosen to resemble the actual leaf temperatures measured with the LI-6800 portable photosynthesis system right before conducting the light response curves. The built-in LI-190 PAR light sensor was used to determine the ambient light intensity at all leaf positions upon measurement of the light response curves. It was ensured that the upper position was similar for all replicates, and that the lower positions were 50–100 μmol m^−2^ s^−1^ PAR lower than the upper position. The relatively large difference in light intensities between positions (Table 2) resulted from large differences between plant stature and growth of leaves at different heights between temperature treatments and species. Relative differences in light intensity between leaf positions as well as at upper leaf positions are shown in Table 2.

**Table 2.** Light intensities at three leaf positions in *Lythrum salicaria* and *Iris pseudacorus* grown at two temperatures. ‘Upper’, ‘Middle’ and ‘Lower’: light intensities at upper, middle and lower leaf position, respectively. Δ _Upper − middle_: average difference in light intensity between upper and middle leaf positions, Δ _Upper − lower_: average difference in light intensity between upper and lower leaf positions. Light intensities are given as mean ± standard deviation.

	Light intensity (μmol m^−2^ s^−1^ PAR)			
	*I. pseudacorus*		*L. salicaria*	
Leaf position	15 °C	25 °C	15 °C	25 °C
Upper	348.8 ± 42.6	283.4 ± 76.6	393.1 ± 37.0	290.0 ± 55.2
Middle	335.1 ± 38.0	256.0 ± 72.3	348.1 ± 54.9	204.8 ± 75.3
Lower	214.1 ± 69.3	103.0 ± 65.9	197.5 ± 42.0	175.2 ± 52.5
Δ _Upper − middle_	13.7	27.4	45.0	85.2
Δ _Upper − lower_	134.7	180.4	195.6	114.8

For *I. pseudacorus*, which had parallel leaves, the measured leaf area was determined using a ruler and entered into the gas analyser prior to measuring light response curves for each leaf position. For *L. salicaria*, which has lanceolate leaves, the leaf area was set at a constant value (3 cm^2^) on the gas analyser and corrected afterwards by harvesting the leaf and determining the exact leaf area, which had been inside the chamber, with a LI-3100 area meter (Li-COR Biosciences, Lincoln, NE, USA). It was necessary to calculate the corrected photosynthetic rate for each leaf using this area. This was done by entering the corrected areas into to the *Excel* spreadsheet generated by the gas analyser console at the end of each measuring day.

Light response curves were fitted with Excel macros provided as online material by Lobo *et al.* (2013). The model fitting the equation of Prioul and Chartier (1977) was chosen since it provided the lowest error sum of squares (SSE). The equation of the fit is given by equation (1), where *A* = photosynthesis rate, *I* = light intensity, Φ = quantum yield, *R*_d_ = dark respiration and *θ* = convexity constant:

$$\begin{array}{l} A=\frac{\Phi I+\sqrt{{\left(\Phi IA_{max}\right)}^{2}-4\theta \Phi IA_{max}}}{2\theta }-R_{d} \end{array}$$ (1)

A fit was made for each curve and for each leaf position and the following parameters were determined: maximum light-saturated photosynthesis rate (*A*_max_), quantum yield (Φ), light compensation point (*I*_c_)_,_ light saturation point (*I*_k_) and dark respiration rate (*R*_d_).

### Specific leaf area

After completing each light response curve, the area of the leaf that had been inside the gas analyser chamber was marked with yellow tape. The leaves were harvested and the marked areas were excised and measured with a leaf area meter (LI-3100, Li-COR Biosciences, Lincoln, NE, USA). The leaf segments were then wrapped in aluminium foil and stored in a freezer at −18 °C. Throughout this procedure, a relatively large proportion of the total biomass of each *I. pseudacorus* replicate was harvested after completing the light response curves, due to the nature of the growth form of this species. The remainder of the harvested leaf was therefore dried in a drying oven for 48 h at 60 °C and stored in a desiccator to later be added to the species’ above-ground biomass. All frozen leaf segments were freeze-dried for 24 h and stored in a desiccator until weighing. The leaf segment area was divided by its dry weight to determine specific leaf area (SLA) as described by Perez-Hardguindeguy *et al.* (2013).

### Photosynthetic pigments and carbon and nitrogen concentrations of leaves

The leaf segments from which SLA was determined were ground in a ball mill (MM 400, Retsch, Haan, Germany). Approximately 5 mg of ground leaf was extracted in 96 % ethanol, and the concentrations of total chlorophyll (Chl_a+b_), Chl_a_, Chl_b_ and total carotenoids (C_x+c_) in the extract were measured by spectrophotometry according to Lichtenthaler (1987). Carbon (C) and nitrogen (N) concentrations in the same ground leaf material were measured with an elemental analyser (Fisons Instruments, Model NA2000, Rodano, Milan, Italy).

### Statistical analyses

The experiment was a 2 × 2 fully factorial design, with the factors ‘Temperature’ (low- and high-temperature treatment, respectively, 15 °C and 25 °C) and ‘Species’ (*L. salicaria* and *I. pseudoacorus*). For light response-related factors and SLA, an additional factor (leaf position; upper, middle and lower) was considered.

Four light response curves showed negative values or zero for *R*_d,_ probably due to very low rates of gas exchange. These were all curves for ‘lower leaf position’ in *L. salicaria* (two replicates in low temperature and two replicates in high temperature). These curves and their related parameters were excluded in the statistical analysis of photosynthetic parameters as well as from the average values of these parameters. Hence, *n* was six rather than eight for the photosynthetic parameters in those treatments.

A Levene’s test for homogeneity of variance was conducted for all growth, photosynthetic and leaf element-related parameters. Above-ground biomass, C/N and chlorophyll/carotenoid ratios were log_2_ transformed in order to satisfy homoscedasticity.

For shoot height and above-ground biomass a two-way analysis of variance (ANOVA) was conducted. Here, the main factors were temperature (high or low) and species (*L. salicaria* or *I. pseudacorus*). The interaction term ‘temperature × species’ was included in the ANOVA. It was ensured that all plants were initially of similar height by comparing the initial heights of the seedlings within species between temperature treatment groups by using an *F*-test and Student’s *t*-test. The initial heights were measured from the top of the pot to the top of the plant. The tests showed no significant differences in initial height between within-species treatment groups. Thus, any differences in final shoot height (within species, between temperature treatments) were a consequence of the temperature treatment.

For all photosynthetic and leaf element-related parameters as well as SLA, a three-way ANOVA was conducted. Here, the fixed main factors were temperature (high or low), species (*L. salicaria* or *I. pseudacorus*) and leaf position (upper, middle or lower). All possible interaction terms were included. Type 3 sum of squares were used since, by the exclusion of four replicates, the three-way factorial design was unbalanced. Finally, a Tukey’s honestly significant differences test was used to determine the differences between treatment groups for all parameters. All statistical analyses were performed with a 5 % significance level using RStudio (RStudio Team 2016; RStudio: Integrated Development for R. RStudio, Inc., Boston, MA, USA).

## Results

### Growth parameters

**Figure 1.**
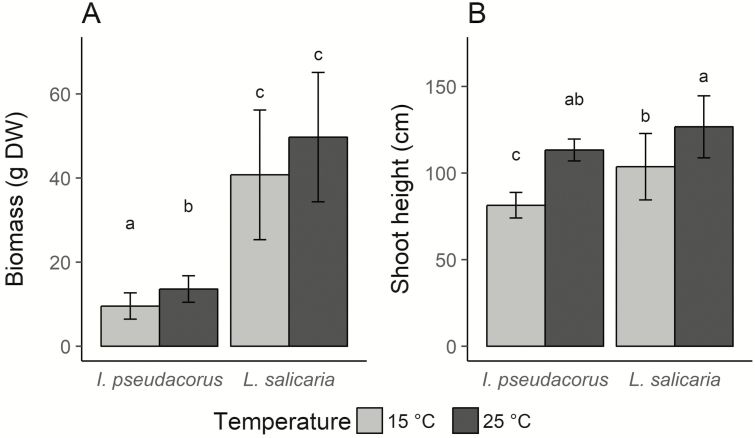
Growth parameters of *Iris pseudacorus* and *Lythrum salicaria*. Mean ± standard deviation of above-ground biomass (A) and final shoot height (B) of both species grown at 15 °C and 25 °C, respectively. Different letters indicate statistically significant differences between groups provided by Tukey’s honestly significant differences test at a 0.05 significance level. DW: dry weight.

Species and growth temperature had a significant effect on both above-ground biomass and shoot height. However, no significant interaction between species and growth temperature was found for these two growth parameters (Table 3). *Lythrum salicaria* had significantly higher above-ground biomass and shoot height than *I. pseudacorus* (Fig. 1A and B). Both species produced the greatest biomass and tallest shoots in the high-temperature treatment (Fig. 1A and B). *Iris pseudacorus* showed a stronger response to increased temperature for both parameters compared to *L. salicaria. Iris pseudacorus* had 42.31 % higher biomass and 39.20 % taller shoots at 25 °C compared to 15 °C, while *L. salicaria* only had 22.02 % higher biomass and 22.16 % taller shoots. We observed that all individuals of *L. salicaria* at 25 °C were flowering, compared to 15 °C, where only 50 % of the replicates were flowering, although phenological traits were not quantified. No flowers were produced in *I. pseudacorus*.

**Table 3.** *F*-values of ANOVA for growth and photosynthetic parameters. Degrees of freedom indicated as ‘df’. Species: *Iris pseudacorus* or *Lythrum salicaria.* Temperature: 15 °C or 25 °C. Leaf position: upper, middle or lower. BM: above-ground biomass, SH: shoot height, *A*_max_: maximum photosynthesis rate, *R*_d_: dark respiration rate, *I*_c_: light compensation point, Φ: quantum yield, *I*_k_: light saturation point, SLA: specific leaf area, Chl_a+b_: total chlorophyll concentration, C_x+c_: total carotenoid concentration, Chl_a+b_/C_x+c_: chlorophyll/carotenoid ratio, Chl_a_/Chl_b_: chlorophyll a/b ratio, N: leaf nitrogen concentration, C: leaf carbon concentration, C/N: leaf carbon/nitrogen ratio, PNUE: photosynthetic nitrogen use efficiency. Significant explaining factors are marked with boldface. Level of significance is marked with asterisks (*P < 0.05; **P < 0.01; ***P < 0.001).

	Source of variation			Interactions			
Parameter	Species (df = 1)	Temperature (df = 1)	Leaf position (df = 2)	Species × temperature (df = 1)	Species × leaf position (df = 2)	Temperature × leaf position (df = 2)	Species × temperature × leaf position (df = 2)
BM	137.919***	6.778*	–	0.384	**–**	–	–
SH	12.966**	30.784***	–	0.815	**–**	–	–
*A*_max_	36.082***	3.525	5.741**	0.00840	0.887	1.977	0.379
*R*_d_	1.389	0.124	0.441	0.0829	2.884	0.0327	1.883
*I*_c_	2.432	0.188	0.864	0.173	6.961**	0.296	0.816
Φ	21.251***	0.00120	5.722**	1.053	2.744	0.337	1.549
*I*_k_	19.159***	0.898	6.667**	0.0280	4.246*	0.715	0.0536
SLA	5.768*	7.691**	3.805*	2.324	20.149***	0.281	2.857
Chl_a+b_	110.333***	36.756***	5.740**	2.248	8.186***	6.931**	1.691
C_x+c_	100.039***	10.258**	4.204*	0.109	3.954*	5.200**	1.391
Chl_a+b_/C_x+c_	15.239***	6.612*	3.074	1.116	0.620	0.448	0.356
Chl_a_/Chl_b_	0.894	20.174***	0.876	0.0262	2.837	0.683	1.138
N	71.085***	2.273	4.602*	0.223	3.871*	1.297	0.133
C	16.209***	2.460	9.677***	3.190	3.788*	0.652	0.0485
C/N	76.090***	1.870	2.138	1.168	3.312*	0.757	0.275
PNUE	4.580*	9.493**	4.426*	1.082	3.248*	1.487	1.465

### Photosynthetic parameters and SLA

**Figure 2.**
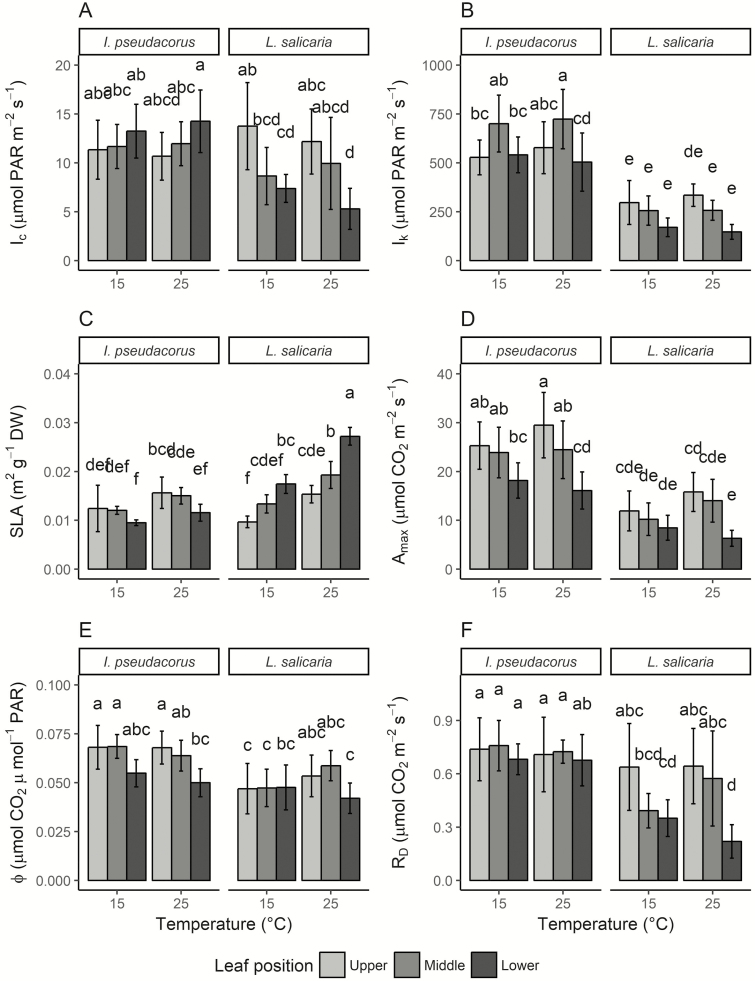
Photosynthetic parameters from light response curves of *Iris pseudacorus* and *Lythrum salicaria.* Mean ± standard deviation of light compensation point *I*_c_ (A), light saturation point *I*_k_ (B), specific leaf area SLA (C), light-saturated photosynthetic rate *A*_max_ (D), quantum yield Φ (E) and dark respiration rate *R*_d_ (F) of both species grown at 15 °C and 25 °C, and measured at three different leaf positions, respectively. Different letters indicate statistically significant differences between groups provided by Tukey’s honestly significant differences test at a 0.05 significance level.

No significant interactions between species and temperature, temperature and leaf position or a three-way interaction were found in any of the measured parameters (Table 3). A significant interaction between species and leaf position was found for *I*_c_, *I*_k_ and SLA (Table 3). In *L. salicaria*, *I*_c_ was lowest in the lower and highest in the upper leaf positions, while it remained more similar across leaf positions in *I. pseudacorus*, although slightly increasing with decreasing leaf position (Fig. 2A). The light saturation point was highest in the middle leaf position in *I. pseudacorus* while it remained similar, albeit somewhat decreasing from upper to lower leaf position, in *L. salicaria* (Fig. 2B). The two species showed opposite responses in SLA across leaf positions. Here, *I. pseudacorus* had a lower SLA in the lower leaf positions while *L. salicaria* had a higher SLA in the lower leaf positions (Fig. 2C). Temperature did not have a significant effect on any of the physiological parameters except SLA, which was generally higher in the high-temperature treatment for both species (Table 3; Fig. 2C).

Species and leaf position had a significant effect on *A*_max_ and Φ but no significant interaction was found (Table 3). Both *A*_max_ and Φ were significantly higher in *I. pseudacorus* than in *L. salicaria*. Overall, upper leaf positions had a higher *A*_max_ and Φ than lower leaves in both species, although Φ in *L. salicaria* at low temperature was similar among leaf positions (Fig. 2D and E).

Dark respiration rate was not significantly affected by any of the factors included in the ANOVA (Table 3). However, the interaction term ‘species × leaf position’ had *P* = 0.0617 which was close to the significance threshold of 0.05, and Tukey’s honestly significant differences test indicated a trend of similar *R*_d_ among leaf positions in *I. pseudacorus* but decreasing *R*_d_ with lower leaf position in *L. salicaria* (Fig. 2F). Dark respiration rates were overall highest in *I. pseudacorus*.

**Figure 3.**
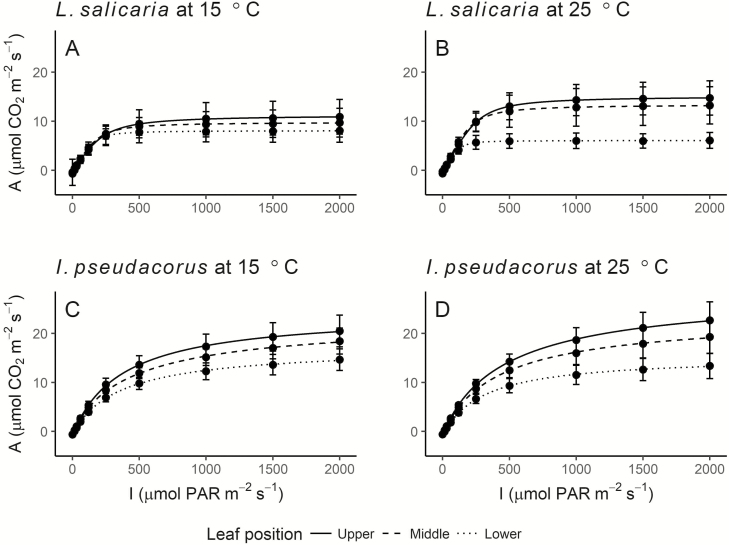
Light response curves of *Iris pseudacorus* and *Lythrum salicaria*. Mean ± standard deviation photosynthetic rates, *A*, measured at different light intensities, *I*, of PAR for *L. salicaria* at 15 °C (A), *L. salicaria* at 25 °C (B), *I. pseudacorus* at 15 °C (C) and *I. pseudacorus* at 25 °C (D), measured at three different leaf positions, respectively. Data were fitted using a nonlinear hyperbolic regression.

Compared to each other, the leaf positions had similar light responses in both species, with upper leaves reaching higher assimilation rates (*A*) at high light intensity than middle leaves, which again reached higher *A* at high light intensity than lower leaves (Fig. 3). Upper and middle leaf positions generally had similar *A*, whereas lower leaves had overall lower *A*. The observed pattern was especially obvious in *L. salicaria* at high temperature. Standard deviations indicate that these observations might not be significant in most cases.

The shape of the curves was very different between the species. *Lythrum salicaria* leaves showed a steep increase in *A* with increasing light intensity from 0 to 250 μmol PAR m^−2^ s^−1^ which was followed by saturation of the curve with fairly constant *A* at light intensities above 500 μmol PAR m^−2^ s^−1^ (Fig. 3A and B). *Iris pseudacorus* leaves showed the same initially steep increase in *A* with increasing light intensity from 0 to 250 μmol PAR m^−2^ s^−1^. However, this was followed by a softer flattening of the curve than for *L. salicaria*, and the curve continued increasing at the higher light intensities without reaching saturation at 2000 μmol PAR m^−2^ s^−1^ (Fig. 3C and D).

### Photosynthetic pigments and leaf C and N concentrations

**Figure 4.**
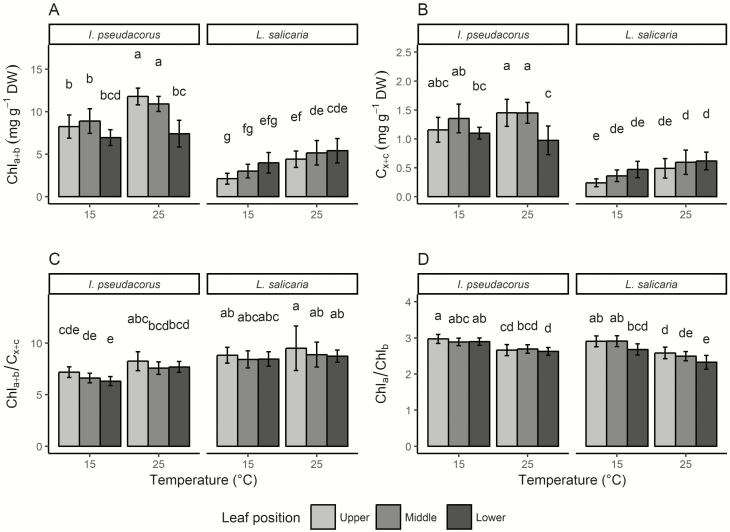
Photosynthetic pigments of *Iris pseudacorus* and *Lythrum salicaria*. Mean ± standard deviation of total chlorophyll concentration Chl_a+b_ (A), total carotenoid concentration C_x+c_ (B), chlorophyll/carotenoid ratio Chl_a+b_/C_x+c_ (C), chlorophyll a/b ratio Chl_a_/Chl_b_ (D) of both species grown at 15 °C and 25 °C, and measured at three different leaf positions; upper, middle and lower, respectively. Different letters indicate statistically significant differences between groups provided by Tukey’s honestly significant differences test at a 0.05 significance level. DW: dry weight.

Total chlorophyll (Chl_a+b_) and total carotenoid (C_x+c_) concentrations were significantly affected by species, temperature and leaf position. Furthermore, significant interactions were found between species and leaf position and between temperature and leaf position (Table 3). In both species, total chlorophyll and carotenoid concentrations were highest at high temperature, but in some cases mainly at specific leaf positions (Fig. 4A and B). In *L. salicaria*, chlorophyll and carotenoid concentrations increased with lower leaf position at both temperatures. In *I. pseudacorus*, the opposite pattern was observed, where ‘lower’ leaf position had the lowest pigment concentrations (Fig. 4A and B). The chlorophyll/carotenoid (Chl_a+b_/C_x+c_) ratio was significantly affected by species and temperature but no significant interactions were found (Table 3). Chlorophyll/carotenoid ratios were higher in *I. pseudacorus* than in *L. salicaria*, and a higher ratio was observed at high temperature in both species. Although the factor ‘leaf position’ had no significant effect on Chl_a+b_/C_x+c_ according to the ANOVA results, a high *F*-ratio with *P* = 0.0517 was found, very close to the significance threshold of *P* < 0.05. Furthermore, Tukey’s honestly significant differences test showed a trend that Chl_a+b_/C_x+c_ decreased with lower leaf position in both species (Fig. 4D). Chlorophyll a/b ratio was only affected significantly by temperature (Table 3), with higher Chl a/b at the low temperature.

**Figure 5.**
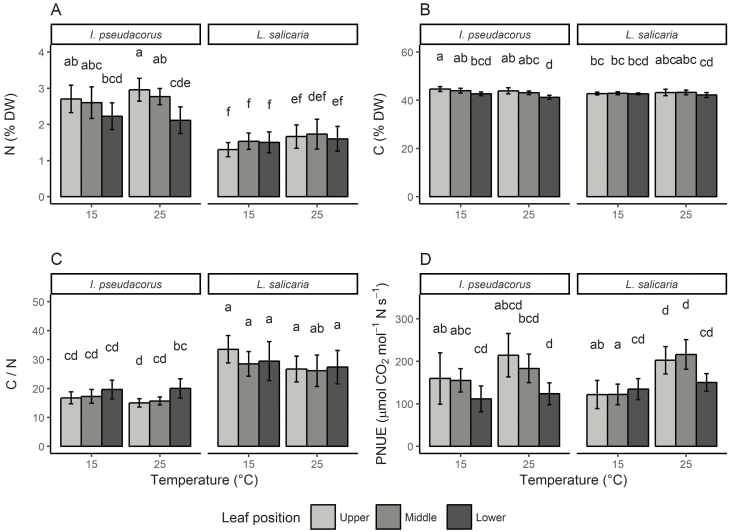
Carbon and nitrogen concentration of *Iris pseudacorus* and *Lythrum salicaria*. Mean ± standard deviation of leaf nitrogen concentration N (A), leaf carbon concentration C (B), leaf carbon/nitrogen ratio (C), photosynthetic nitrogen use efficiency PNUE (D) of both species grown at 15 °C and 25 °C, and measured at three different leaf positions (upper, middle and lower), respectively. Different letters indicate statistically significant differences between groups provided by Tukey’s honest significant differences test at a 0.05 significance level. DW: dry weight.

Leaf N, C and C/N ratio were significantly affected by species, leaf position and their interaction (Table 3). Both C and especially N concentrations were highest in *I. pseudacorus*, but decreased with lower leaf position, while remaining similar among leaf positions in *L. salicaria* (Fig. 5A and B). Leaf C/N ratio was 34–104 % higher in *L. salicaria* than in *I. pseudacorus* (Fig. 5C). In *I. pseudacorus*, the C/N ratio increased with lower leaf position, in particular at the high temperature, whereas the C/N ratio of *L. salicaria* was similar between leaf positions at both temperatures. Photosynthetic nitrogen use efficiency (PNUE) was significantly affected by species, temperature, leaf position and ‘species × leaf position’ (Table 3; Fig. 5D). In general, PNUE was higher at high temperature than at the low temperature. While PNUE of *I. pseudacorus* decreased with lower leaf position, in *L. salicaria* PNUE was similar between leaf positions and only lower at the lowest leaf position in the high temperature.

## Discussion

This study aimed to understand gas exchange, growth, pigment and C/N-related responses in *L. salicaria* and *I. pseudacorus* to different temperature regimes in order to build an understanding of temperature acclimation in invasive plants with different morphology. *Iris pseudacorus* has vertical leaves which provide minimal self-shading while *L. salicaria* has horizontal leaves and extensive self-shading. We conducted a comparative analysis of the two species exposed to high (25 °C) and low (15 °C) growth temperature treatments. Remarkably, temperature did not significantly affect any of the gas exchange-related traits (*A*_max_, Φ, *I*_c_, *I*_k_, *R*_d_). With a temperature difference as large as 10 °C, an effect on these traits would have been expected, since gas exchange is often affected by temperature, even though this can vary among species (Turnbull *et al.* 2002; Niu *et al.* 2008). The absence of temperature effects on gas exchange-related traits suggests that the thermal range chosen for our experiment was well within the natural range in which both species can function normally, and that both species are relatively plastic, since they were able to maintain constantly high photosynthesis rates across the temperature range used (Yamori *et al.* 2005).

We found that leaf position in the canopy had a much stronger effect on gas exchange than temperature. Quantum yield and especially *A*_max_ decreased with lower leaf position in both species. The effect of leaf position was most pronounced in the high-temperature treatment and in *L. salicaria*, where the lower leaf position had a very low *A*_max_ compared to middle and upper leaf positions. As light intensity is attenuated through the canopy, photosynthetic rates and *A*_max_ can be expected to decline (Björkman 1981; Hirose and Werger 1987). A typical response to low light availability is an increased investment in light-harvesting-associated pigments (Lichtenthaler *et al.* 1981; Melis and Harvey 1981). This strategy was indeed used by *L. salicaria*, which allocated more Chl_a+b_ to lower leaves, as opposed to *I. pseudacorus*. Hence, while low *A*_max_ in lower leaf positions can partly be attributed to less acclimation of light harvesting in *I. pseudoacorus*, this cannot fully explain the response in *L. salicaria*.

Another shade acclimation strategy of *L. salicaria* was allocation of similar amounts of N to all leaf layers and maintenance of similar C/N ratios. Nitrogen concentration in leaves can serve as a proxy for investment in photosynthetic enzymes (Evans 1989; Poorter and Evans 1998; Sun *et al.* 2016) and, especially at low temperature, this investment lead to sustained PNUE in *L. salicaria.* However, PNUE of the lower leaf was reduced at 25 °C, which suggests either an overinvestment of N or N allocation to other than assimilation-related structures, since it was not met by a similarly maintained photosynthesis (Onoda *et al.* 2004). In *I. pseudacorus*, N allocation as well as PNUE was reduced with decreasing leaf position. Also, higher chlorophyll and carotenoid concentrations were observed in upper and middle leaf positions, suggesting that *A*_max_ was maximized in upper and middle leaf positions by increased concentrations of photosynthetic pigments and N allocation. *Lythrum salicaria* acclimated to reduced light availability by increasing photosynthetic pigment concentrations, the relative amount of Chl_b_, which is mainly associated with photosynthetic light-harvesting complexes (Hoober *et al.* 2007), and by maintaining high leaf N concentrations in the lower part of the canopy.

Specific leaf area also reflected the different light acclimation strategies of the two species, as SLA of *L. salicaria* increased with lower leaf positions while decreasing in *I. pseudacorus*. Leaves with high SLA are generally thinner than leaves with low SLA, due to fewer structural components. Thin leaves are less costly to produce (Daehler 2003), which can allow the plant to invest resources in other structures, such as flowers, enhancing dispersal. Thinner leaves also allow for a more efficient gas diffusion over the leaf surface, ultimately favouring higher rates of photosynthesis (Flexas *et al.* 2012). Moreover, the different growth forms of the two species can explain the differences in SLA. The lower parts of the leaves in *I. pseudacorus* are likely to contain relatively large amounts of structural leaf components in order to support the long vertical leaf. This trend has been found in grasses which have similar leaf shape (Ocheltree *et al.* 2012). In *L. salicaria*, the same is not necessary since the leaf is attached to the stem providing structural support. Thinner leaves are a typical acclimation to shade, allowing for a higher light penetration (Blondeel *et al.* 2020). Shading was more pronounced for lower leaves of *L. salicaria* grown at 25 °C than at 15 °C, which was reflected in the species’ SLA over the canopy.

In general, and unlike *L. salicaria*, *I. pseudacoris* showed no clear signs of shade acclimation in lower leaf positions. The light response curves showed that *I. pseudacorus* did not reach complete light saturation in any leaf position even at 2000 μmol PAR m^−2^ s^−1^, revealing that this species is well-adapted to high-light environments throughout its canopy. Such a phenomenon is usually observed in C4-photosynthetic species, but C3 species, like *I. pseudacorus*, can well have high assimilation rates and productivity, due to high inherent photosynthetic traits like carboxylation rate and electron transport rate (Webster *et al.* 2016). The overall higher chlorophyll, carotenoid and N concentrations of *I. pseudacorus* compared to *L. salicaria* suggested relatively larger amounts of photosynthetic enzymes and chloroplasts (Evans 1989; Sun *et al.* 2016). However, net assimilation rates of light response curves showed stronger differences at different leaf positions in *I. pseudacorus* than *L. salicaria*, as a stronger decrease in C assimilation was a response by the less shade-acclimated, lower leaf positions in *I. pseudacorus*.

Long leaves, such as those of *I. pseudacorus* are partitioned during leaf development, with the growing base and the aging tip functioning as sinks to photosynthetic products and the middle of the leaf as transition zone from sink to source (Li *et al.* 2010). While we tried to ensure to cover a light gradient on leaf parts of similar age by measuring on similar green areas, we cannot completely rule out a certain age effect over the three leaf positions on *I. pseudacorus*. However, our light response results strongly suggest that light differences were the main regulating factor, not age. Otherwise, the middle position would have yielded the highest photosynthetic activity, which was not the case. Also, we would have detected much higher respiration rates in the lower leaf position, due to high metabolic activity in developing leaf parts (Li *et al.* 2010).

The cardinal points derived from the light response curves reveal further differences in photosynthetic light acclimation strategies between the two species. Thus, the light compensation point (*I*_c_) increased with lower leaf position in *I. pseudacorus* while it decreased in *L. salicaria*. Light compensation point defines the light intensity at which photosynthesis and dark respiration (*R*_d_) are balanced, and net assimilation rates are zero. *Iris pseudacorus* had similar *R*_d_ at all leaf positions while photosynthesis decreased towards the bottom of the canopy. Hence, net C gain was greater in the upper canopy compared to the lower canopy where respiration was higher relative to photosynthesis rate. In contrast to this, *L. salicaria* had decreasing Φ, *R*_d_ and *A*_max_ towards the bottom of the canopy, resulting in decreasing *I*_c_, a typical acclimation to shading (Herrmann *et al.* 2020). Reduced *R*_d_ in shade leaves is symptomatic for a lower metabolic activity and decreased need for protection from high irradiance (Plaxton 1996; Herrmann *et al.* 2020). Morphologically, *I. pseudacoris* is potentially exposed more often to higher radiation in the lower canopy, due to its lanceolate leaves, in contrast to *L. salicaria*, in which leaves at the bottom of the canopy are mostly self-shaded. Hence, higher *R*_d_ in *I. pseudacorus* may facilitate protection from solar radiation and support the lower SLA and higher structural support needed. Some caution of interpreting C gain based on *A*_max_ and *R*_d_ is, however, advised. Day respiration in light is not necessarily equal to *R*_d_, since part of the CO_2_ released during daytime, by day respiration or photorespiration, can be re-captured within the photosynthetic cell (Tcherkez *et al.* 2017). Also, day respiration is inhibited in the light (Heskel and Tang 2018; Keenan *et al.* 2019). There is no convenient, easily implemented and accurate enough method of determining day respiration (Tcherkez *et al.* 2017). However, the potential bias resulting especially from light inhibition of day respiration would only emphasize our findings: in high light at the top of the canopy, respiration rates may in fact have been lower and C gain higher during the day, while that would not have been the case in a self-shaded canopy.

The light saturation point (*I*_k_) is defined as the light intensity where assimilation rates become less limited by electron transport rates and more by Calvin Cycle reactions (Herrmann *et al.* 2020). As *I*_k_ was highest in middle leaf positions in *I. pseudacorus*, this species had the highest light demand until approaching light saturation in the middle of its canopy, providing evidence that it is indeed adapted to high-light environments with minimal shading. Contrastingly, decreasing *I*_k_ over the canopy of *L. salicaria*, and the distinct light-saturated phase of the species’ light response curves, demonstrated that especially the lower leaves of *L. salicaria* were adapted to low light intensities (Dias-Filho 1997, 2002). The shading that *L. salicaria* is exposed to may be primarily provided by its own canopy since this species is often found in light-open habitats, for example on river banks (Mitich 1999).

No statistical evidence was found that *L. salicaria* was better adapted to low than high temperatures with regard to gas exchange traits. Different metabolic acclimation strategies, such as regulation of the electron transport capacity or the capacity for storage of carbon compounds, may lead to similar photosynthetic responses even under different growth temperatures, as long as those conditions are not extreme (Herrmann *et al.* 2020). Nonetheless, we detected a tendency for negative warming impacts on the lower leaves grown at 25 °C, which was evident in considerably lower light response curves, Φ, *R*_d_, *A*_max_ and PNUE. This finding was in accordance with our expectation that light attenuation would affect gas exchange of *L. salicaria* more than *I. pseudacorus*. Generally, photosynthetic responses to different light availability seem to be similar in species with similar morphology, even in closely related species from contrasting habitats (da Costa *et al.* 2019). Moreover, warming resulted in higher SLA, especially in *L. salicaria*. Leaves in the high-temperature treatment were therefore thinner than in the low-temperature treatment, which could possibly be explained by faster growth rates in leaves with fewer structural components, allowing a larger investment of resources in other structures, such as flowers (Perez-Hardguindeguy *et al.* 2013). We indeed observed more flowering individuals of *L. salicaria* at 25 °C compared to 15 °C.

Opposite to our expectation, no evidence was found that *I. pseudacorus* was more susceptible to photoinhibition at low temperature than at high temperature, as there was no significant difference in *A*_max_ and Φ between temperatures. A likely explanation could be effective quenching of excess light energy, such as chlorophyll fluorescence, ridding the photosynthetic apparatus of excess excitation energy, and preventing photoinhibition in the species (Powles 1984; Jespersen *et al.* 2017; Sello *et al.* 2019). The shape of the light response curves for *I. pseudacorus* was very similar across temperature treatments even though curves were slightly flatter at high light intensities in the low temperature, at least for the upper leaf position. Nonetheless, *I. pseudacorus* had higher biomass production and greater final shoot height in the high temperature, compared to the low temperature. In addition, its chlorophyll concentration and PNUE were higher at high temperature, indicating a photosynthetic apparatus adapted to warm, high-light environments (Webster *et al.* 2016). *Lythrum salicaria* was less responsive to temperature than *I. pseudacorus* in its vegetative growth, but its flower development was considerably enhanced unlike growth at 15 °C. Hence, although the lower leaves of the species at 25 °C indeed seemed to have lower assimilation rates, confirming our expectation, the overall performance of *L. salicaria* was positively affected by the higher temperature.

Both species are therefore capable of developmental acclimation and likely to follow the general pattern of shifting habitats to higher latitudes and elevations in a warming climate (Athanasiou *et al.* 2010). *Lythrum salicaria* in Canada is known to be limited in its northern distribution by cold temperatures (Thompson *et al.* 1987), and with temperatures rising most dramatically in the Arctic and subarctic regions, its range is likely to extend rapidly to the north. Moreover, faster flowering development may increase the invasive hazard of the species as the climate warms. A range extension to the north is also likely for *I. pseudacorus* with rising temperatures, both in Canada and Scandinavia, as the species showed increased growth at high temperature. Management of both invasive species should take this forecast into account.

In conclusion, our study indicated that increased temperature within the range used in this experiment will benefit these two species, despite their different morphology, and even though their photosynthetic traits were less affected. Light response curves revealed that *L. salicaria* allocated more photosynthetic pigments and N to lower leaf positions than *I. pseudacorus*, and was therefore better acclimated to low light intensities than *I. pseudacorus.* Both species are usually found in the same light-open type of habitat. We therefore suggest that *L. salicaria* showed an adaptive response to self-shading in its canopy. Both species should continue to be recognised as highly invasive under future climate scenarios in management plans, and the impending advancement of their northern ranges seen as a threat to the health of native ecosystems.

## Supporting Information

The following additional information is available in the online version of this article—File S1. All data necessary for the reproduction of this study. Infrared gas analyser (IRGA) data for all leaf positions of *Lythrum salicaria* and *Iris pseudacorus* used for light response curves and determination of photosynthetic parameters (sheet 2 + 3). Pigment content of all leaf positions of *L. salicaria* and *I. pseudacorus* (sheet 4). Carbon and nitrogen content of all leaf positions of *L. salicaria* and *I. pseudacorus* (sheet 5). Areas and dry weight of all harvested leaf segments (sheet 6). Aboveground biomass and shoot height of all plants (sheet 7). Photosynthetic parameters derived from light response curves as well as SLA (specific leaf area) for all leaf positions in *L. salicaria* and *I. pseudacorus*.

## Supplementary Material

plaa031_suppl_Supplementary_File_S1

plaa031_suppl_Supplementary_Data
